# Holographic Formation of Non-uniform Diffraction Structures by Arbitrary Polarized Recording Beams in Liquid Crystal-photopolymer Compositions

**DOI:** 10.3390/polym11050861

**Published:** 2019-05-11

**Authors:** Artem Semkin, Sergey Sharangovich

**Affiliations:** Department of Microwave and Quantum Radio Engineering, Tomsk State University of Control Systems and Radioelectronics, 634050, Tomsk, Russia; shr@tusur.ru

**Keywords:** holographic polymer-dispersed liquid crystals, polymer-stabilized liquid crystals, holography, diffraction gratings, polarization diffraction gratings

## Abstract

In this work, the theoretical model of non-uniform diffraction structures’ holographic formation in liquid crystal-photopolymer (LC-PPM) composite materials with a dye-sensitizer is developed. The model takes into account the arbitrary character of amplitude and phase spatial distributions of recording light field, its arbitrary polarization state and also a non-linearity of the recording process. Two the most common types of liquid crystal-photopolymer composite are investigated: Holographic polymer-dispersed liquid crystals (H-PDLC) and polymer-stabilized liquid crystals (PSLC). Numerical simulations for the most common cases of holographic formation schemes are made. It is shown that due to the photo-induced Freedericksz transition, in the case of arbitrary polarization states of recording light beams, the non-uniform polarization diffraction grating (PDG) is formed in LC-PPM. Numerical simulations’ results show that PDG’s contribution to the change of the dielectric tensor of the media is comparable with the contribution of the photopolymerization-diffusion process.

## 1. Introduction

Diffraction structures holographically formed in composite photopolymer materials (containing monomer, dye, initiator, plasticizer, etc.) are well known since 1960s–1970s [1,2,3]. In the same period, H. Kogelnik published an article [4] with the coupled waves theory for the light diffraction on bulk phase gratings. The first theoretical models of diffraction structures’ holographic formation in such media appeared somewhat later [5,6].

The holographic diffraction structure (HDS) is formed by the light field’s impact on the photo-sensitive photopolymer media. Formation field has the form of a periodic distribution of illuminated and unlit areas (interference pattern). In the lighted areas under the action of radiation the photopolymerization process proceeds. Before the exposure, the composition was a homogeneous mixture of components, a gradient of concentrations of polymer, and monomer is formed in the media volume. It leads to diffusion of the monomer into the lighted areas and crowding out the components, not involved in the reaction, into the unlighted areas [7].

Photopolymer compositions containing molecules of liquid crystals (LCs) are of particular interest, since LCs’ presence in composition causes the anisotropy of its optical properties, and also allows observing electro-optical orientation effects that are common for LCs [8]. All known liquid crystal-photopolymer (LC-PPM) composites can be divided into several types [8,9]. Two of them are investigated in this article as the most commonly used:

1. Polymer-dispersed liquid crystals (PDLC) [10,11]. It is typical for these materials to combine LC molecules into droplets.
2. Polymer-stabilized liquid crystals (PSLC) [12,13,14,15,16]. For these materials, due to various reasons, the process of combining LC molecules into droplets is not typical.

The main difference (essential for the present study) between described compositions is the set of orienting forces acting on LC molecules. In the PDLC samples, the orientation of the molecules in the droplet is determined by the surrounding polymer chains [17]. In the samples of PSLC, the orientation of the LC molecules is mainly determined by the adhesion forces with the bounding surfaces (the substrate of the sample) [18].

Nowadays, a new type of displays for augment reality (AR) technologies, head mounted displays, and other types of see-through displays are rapidly developing [19,20,21]. It was shown (for example in Reference [16]), that it is possible to create multi-plane switchable display on the base of liquid crystal-photopolymer composite materials. Due to the wide range of possible applications of such products and due to complicated structure of them, it is extremely important to develop the theoretical model, taking into account the most complete set of possible effects that determine the key characteristics of the designed devices.

Some theoretical models of HDS formation in photopolymerizable compositions [22,23,24,25,26,27,28], including photopolymer-liquid crystals ones [29,30,31], are developed by the present time.

They consider (among other things):

- the inhomogeneity of the amplitude and phase profile of the recording field;
- photo-induced light absorption changings;
- a considerable degree of nonlinearity of recording process.

In our previous works [32,33,34] we have shown that due to the influence of the photo-induced Freedericksz transition [35,36], it is possible to form the polarization diffraction gratings (PDGs) in LC-PPM by the polarization holography methods.

When designing а complicated holographic device (for example AR display) based on anisotropic liquid crystal-photopolymer composite materials, containing slanted transparent, and/or reflection gratings, the developer must consider the contributions of all of the influencing recording nonlinear processes: Photopolymerization, diffusion, and polarization grating formation.

Thus, the purposes of the present work are:

- to develop a theoretical model, taking into account all of the described effects;
- to numerically investigate the value of PDG’s contribution to the change of the dielectric tensor of the media.

## 2. Theoretical Model

**Figure 1:**
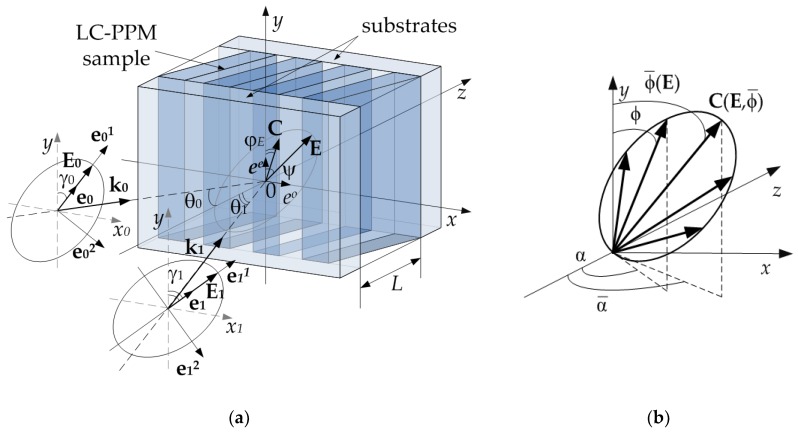
The description of formation of non-uniform HDS in LC-PPM problem: (**a**) Formation geometry; (**b**) the distribution of the LC director in the ellipsoidal droplet.

Schematic image of the investigated sample and formation geometry are shown in Figure 1a. A layer of PSLC or PDLC is placed between two flat parallel plates of an optically transparent material (glass, polymer, etc.). The general case of the incidence of two coherent arbitrarily polarized monochromatic light waves on the interface between the LC-PPM-air is considered.

In Figure 1a, the following notations are introduced: $E_{j}^{}\left(r\right)=A_{j}\left(r\right)\cdot e_{j}\cdot \exp\left[i\cdot \varphi_{j}\left(r\right)\right]$–the electric field vectors of the incident light beams; $A_{j}\left(r\right)$, $\varphi_{j}\left(r\right)$–spatial amplitude and phase profiles of recording beams, respectively; $k_{0}^{}$, $k_{1}^{}$–wave vectors of the recording beams; $e_{j}=\frac{e_{j}^{1}+i\cdot \rho_{j}\cdot e_{j}^{2}}{\sqrt{1+\rho_{j}^{2}}}$–unit complex polarization vectors in their own polarization bases; $e_{j}^{1}$,$e_{j}^{2}$–orts of their own polarization bases; $e_{}^{e}$, $e_{}^{o}$–orts that determine the polarization of extraordinary and ordinary waves, respectively; $\rho_{j}$–ellipticity of polarization ellipses; r–radius-vector; *j* = 0,1; $\theta_{j},\;\gamma_{j}$–angles of incidence and polarization of the incident beams, respectively; E=E(r,t)–vector of the electric field of the light wave in the sample; ψ(r)–angle of rotation of the total light wave polarization; $\varphi_{E}\left(E,r\right)$–angle of rotation of the director of LC molecules (droplets) C=C(r,t) under the action of the photo-induced Freedericksz transition [35,36] in the *xOy* plane.

The optical properties of the samples under study are determined by the dielectric permittivity tensor, which can be determined as follows for the PSLC [30]:

(1) $$\hat{\varepsilon }=(1-\rho )\cdot \varepsilon_{p}\cdot \hat{I}+\rho \cdot {\hat{\varepsilon }}_{lc},$$

where ρ–volume fraction of liquid crystal in the initial composition; $\hat{I}$–unit tensor; $\varepsilon_{p}={\left(n_{p}\right)}^{2}$–dielectric permittivity, $n_{p}$–refraction index of polymer; ${\hat{\varepsilon }}_{lc}=\varepsilon_{lc}^{o}\cdot \hat{I}+(\varepsilon_{lc}^{o}-\varepsilon_{lc}^{e})\cdot CC$–permittivity tensor of LC; $\varepsilon_{lc}^{o}={\left(n_{lc}^{o}\right)}^{2}$, $\varepsilon_{lc}^{e}={\left(n_{lc}^{e}\right)}^{2}$–tensor components measured with longitudinal and transverse orientations of the LC director, respectively; $n_{lc}^{o}$, $n_{lc}^{e}$–ordinary and extraordinary refractive indices of the LC, respectively.

The PDLC dielectric tensor is defined in a similar way [37]:

(2) $$\hat{\varepsilon }=(1-\rho )\cdot \varepsilon_{p}\cdot \hat{I}+\rho \cdot \langle {\hat{\varepsilon }}_{lc}\rangle ,$$

where $\langle {\hat{\varepsilon }}_{lc}\rangle =\varepsilon_{lc}^{o}\cdot \hat{I}+(\varepsilon_{lc}^{o}-\varepsilon_{lc}^{e})\int_{0}^{\pi }\int_{0}^{\pi }CC\cdot p(\alpha )q(\phi )d\alpha d\phi$–statistically averaged LC permittivity tensor, 〈…〉 means a statistical averaging operation; averaging is performed over the angles of orientation of the LC molecules α,ϕ in ellipsoidal droplet (Figure 1b), distributions of which have the form of Gaussian functions [10]:

(3) $$p(\alpha )=A\exp\left[-{\left(\alpha -\bar{\alpha }\right)}^{2}/2\sigma_{\alpha }^{2}\right],$$

(4) $$q(\phi )=B\exp\left[-{\left(\phi -\bar{\phi }\right)}^{2}/2\sigma_{\phi }^{2}\right],$$

where $\bar{\alpha }$, $\bar{\phi }$–average values; $\sigma_{\alpha }^{}$, $\sigma_{\phi }^{}$–standard deviations; A, B–normalization constants that are defined in [10].

Hereinafter, the following notation for vector analysis operations is used:

- A⋅B–scalar product of two vectors A and B;
- A×B–vector product of two vectors A and B;
- AB–tensor product (dyad) of two vectors A and B.

**Figure 2:**
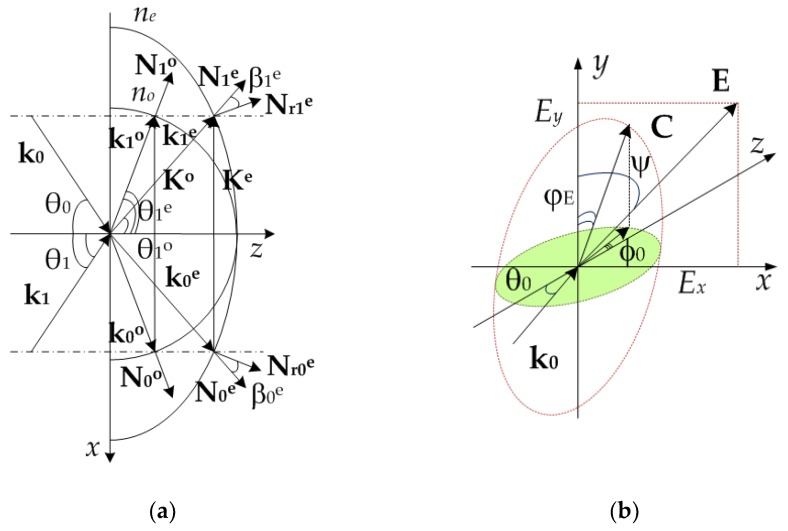
Illustrations of a non-uniform HDS formation: (**a**) Vector diagram for recording of phase diffraction gratings; (**b**) photo-induced Freedericksz transition.

Inside the PSLC (PDLC) sample, recording beams will be attenuated due to non-zero absorption coefficient. Due to the photo-bleaching of dye, the attenuation coefficient will change with time [26]. In addition, due to the anisotropy of the optical properties of the sample, each recording wave inside the sample will generally be divided into two orthogonally polarized ordinary and extraordinary waves. Then the recording waves inside the sample can be described by the following expression:

(5) $$E_{j}^{m}\left(r,t\right)=A_{j}^{m}\left(r\right)\cdot e_{j}^{m}\cdot \exp\left[i\cdot \varphi_{j}^{m}\left(r\right)\right]\cdot \exp\left[-\alpha \left(r,t\right)\cdot \left(N_{rj}^{m}\cdot r\right)\right],$$

where $N_{rj}^{m}$–group normals, their directions differ from wave normals $N_{j}^{m}$ on the angle $\cos(\beta_{j}^{m})=(N_{j}^{m}\cdot N_{rj}^{m})$; α(r,t)– absorption coefficient taking into account its photoinduced change [26]; *m = o* corresponds to own ordinary wave in the sample, *m = e* corresponds to own extraordinary wave in the sample; amplitude distributions in anisotropic media can be defined from equations: $N_{rj}^{m}\cdot \nabla A_{j}^{m}\left(r\right)=0$, its solutions for *xOz* plane can be found in the following way (Figure 2a): $A_{j}^{m}\left(x,z\right)=A_{j}^{m}\left[x-z\tan\left(\theta_{j}^{m}+\beta_{j}^{m}\right)\right]$; $\varphi_{j}^{m}\left(r\right)=\omega t-k_{j}^{m}\left(r\right)\cdot r$–phase spatial distributions; $k_{0}^{m}$, $k_{1}^{m}$–wave vectors of the recording beams (can be dependent on spatial coordinates).

Hereinafter, it is assumed that width of recording beams is much more then thickness of the sample (geometrical optics approximation).

In general case, the distribution of the optical field (5) will form a diffraction structure representing the superposition of five diffraction gratings:

- two phase polymer ones (for ordinary and extraordinary waves), formed due to photo-polymerization processes and inter-diffusion of the components of the media;
- two phase liquid crystal ones (for ordinary and extraordinary waves), formed due only to diffusion processes;
- polarization liquid-crystal one, formed due to the orientation of LC molecules under the action of electrical field of recording waves.

Figure 2a shows a vector diagram for recording of phase diffraction gratings. Figure 2b contains an illustration of the photo-induced Freedericksz transition—the effect of the reorientation of LC molecules under the action of an electric field of an incident light wave [35,36].

In Figure 2 the following notations are additionally introduced: $K_{}^{m}=k_{1}^{m}-k_{0}^{m}$–phase gratings’ vectors; $k_{0}^{m}$, $k_{1}^{m}$–wave vectors of the recording beams; $\phi_{0}$–the refraction angle of $k_{0}^{}$ beam during its propagation in the medium; as before, *m = o* corresponds to own ordinary wave in the sample, and *m = e* corresponds to own extraordinary wave in the sample.

Since the changes in the dielectric permittivity tensor of the PSLC (PDLC) sample, caused by the recording processes, are small relative to the unperturbed state [31,32,33,34], it is possible to present the resulting distribution of perturbed dielectric permittivity tensor in the form:

(6) $$\mathrm{for}\;\mathrm{PSLC}\;–\;\hat{\varepsilon }\left(r,t\right)=(1-\rho )\left[\varepsilon_{p}\cdot \hat{I}+\sum_{m=o,e}^{}\Delta {\hat{\varepsilon }}_{p}^{m}\left(r,t\right)\right]+\rho \left[{\hat{\varepsilon }}_{lc}+\sum_{m=o,e}^{}\Delta {\hat{\varepsilon }}_{lc}^{m}\left(r,t\right)+\Delta {\hat{\varepsilon }}_{lc}^{pol}(r,t)\right],$$

(7) $$\mathrm{for}\;\mathrm{PDLC}\;–\;\hat{\varepsilon }\left(r,t\right)=(1-\rho )\left[\varepsilon_{p}\cdot \hat{I}+\sum_{m=o,e}^{}\langle \Delta {\hat{\varepsilon }}_{p}^{m}\left(r,t\right)\rangle \right]+\rho \left[\langle {\hat{\varepsilon }}_{lc}\rangle +\sum_{m=o,e}^{}\Delta {\hat{\varepsilon }}_{lc}^{m}\left(r,t\right)+\langle \Delta {\hat{\varepsilon }}_{lc}^{pol}(r,t)\rangle \right].$$

where $\Delta {\hat{\varepsilon }}_{p}^{m}\left(r,t\right)$, $\Delta {\hat{\varepsilon }}_{lc}^{m}\left(r,t\right)$, $\Delta {\hat{\varepsilon }}_{lc}^{pol}(r,t)$–changes in the dielectric tensor due to photo-polymerization, diffusion and polarization recording mechanisms respectively.

Thus, to describe the holographic formation of non-uniform diffraction structures every term in the expressions (6, 7) must be defined.

### 2.1. Polarization Recording Mechanism

If in expression (5) the vectors $E_{{}_{j}}^{m}$ are not collinear, then in the sample plane there will be a “polarization” interference pattern formed by the sum of two incident beams. Changing the phase difference between them will change the polarization characteristics of the total light wave. To describe the polarization characteristics of the total optical field, similarly to [32,33,34], the Jones formalism should be applied:

(8) $$J_{j}^{m}(r,t)=A_{j}^{m}(r)\cdot \exp\left\lfloor -\alpha \left(r,t\right)\cdot \left(N_{rj}^{m}\cdot r\right)\right\rfloor \exp\left\lfloor i\cdot \varphi_{j}^{m}(r)\right\rfloor \cdot M_{j}^{m}\cdot R_{j}^{m}\cdot D_{j}^{m},$$

where $D_{j}^{m}$–Jones vectors of recording arbitrarily polarized waves in their own polarization bases; $R_{j}^{m}=\left[\begin{array}{cc} \cos(\gamma_{j}^{m}) & -\sin(\gamma_{j}^{m})\\ \sin(\gamma_{j}^{m}) & \cos(\gamma_{j}^{m}) \end{array}\right]$, $M_{j}^{m}=\left[\begin{array}{cc} \cos(\theta_{j}^{m}) & 0\\ 0 & 1 \end{array}\right]$–matrix of reverse rotation of coordinate axes.

Then the Jones vector of the resulting wave can be found from the expression:

(9) $$J_{}^{m}(r,t)=\sum_{j=0,1}J_{j}^{m}(r,t).$$

To determine the polarization characteristics of the resulting wave a phasor is introduced:

(10) μ(r,t)=Jo(r,t)Je(r,t),

where $J^{o}(r,t)$, $J^{e}(r,t)$–the components of the resulting wave corresponding to its own waves in the sample (ordinary and extraordinary, respectively).

Then the azimuth ψ(r,t) and ellipticity ρ(r,t) distributions of resulting light field can be determined by:

(11) ψ(r,t)=12⋅arctg(2⋅Re[μ(r,t)]1−|μ(r,t)|2), ρ(r,t)2=1−[1+4⋅Im2[μ(r,t)]/(1−|μ(r,t)|2)2]0.51+[1+4⋅Im2[μ(r,t)]/(1−|μ(r,t)|2)2]0.5,

The obtained expressions (8)–(11) completely describe the polarization state of the recording field and its spatial periodic variation. Since the considered materials PSLC and PDLC have morphological features, their interaction with the recording field will be described in different ways. Each composition is considered separately.

According to Reference [35], the dependence of the director C rotation angle on the external electric field value $\varphi_{E}(r,t)$ is determined from the solution of the free energy balance equation. In this case the spatial distribution of electric field of the recording optical wave should be taken into account. This equation (with strong adhesion of LC molecules with bounding surfaces taken into account) has the form [32]:

(12) ∫0φE[sin2ψ(r)−sin2φ′]−12dφ′=1ξe(r,t)(L2±z),

where ξe(r,t)=[K33⋅8πεlce−εlco⋅1E(r,t)2]12–distribution of electrical coherent length; $K_{33}$–Frank coefficient of elasticity; $E(r,t)=\left|\sum_{m=o,e}E_{j}^{m}\left(r,t\right)\right|=\sqrt{I(r,t)}$, I(r,t)–resulting field intensity.

Sign «+» in the expression (12) corresponds to increasing angles LC molecules’ rotation when $0\leq z<\frac{L}{2}$, sign «–» corresponds to decreasing angles when $\frac{L}{2}\leq z\leq L$.

Thus, to determine the distribution of the rotation angle of the LC director under the action of the photo-induced Freedericksz transition, it is necessary to solve two equations (12) at the appropriate intervals. Equation (12) is the initial one for determining the perturbation of the dielectric tensor of the PSLC sample $\Delta {\hat{\varepsilon }}_{lc}^{pol}(r,t)$ through the distribution of the director rotation angle of the liquid crystal $\varphi_{E}(r,t)$.

Further, it is necessary to determine the angle of the droplets rotation in the PDLC under the action of the electric field of the recording wave. The deformation of the quasi-ellipsoidal droplets is not taken into account in this model. The following expression describes the desired angle [38]:

(13) $$\varphi_{E}^{}(r,t)=\psi (r)+\frac{1}{2}\cdot arctg\left[\frac{\sin\left[2\vartheta_{0}(r)\right]}{e^{2}(r,t)+\cos\left[2\vartheta_{0}(r)\right]}\right],$$

where $\vartheta_{0}(r)=\varphi_{0}-\psi (r)$–angle between vectors E and at the initial moment of time (before the recording process); $e(r,t)=E(r,t)R\sqrt{\Delta \bar{\varepsilon }/K_{33}\left(5.7\delta^{2}+2.1\lambda \right)}$–parameter characterizing the effect of an electric field on a bipolar LC droplet; R–droplet radius; δ–droplet eccentricity; $\lambda =RW_{a}/K_{33}$–surface adhesion parameter; $W_{a}$–azimuth surface coupling coefficient; and $\Delta \bar{\varepsilon }$–effective dielectric anisotropy of a bipolar droplet [38].

The critical electric field at which the Freedericksz effect will be observed for PDLC is determined by the expression:

(14) Ec=1R⋅(5.7δ2+2.1WaRΔε¯)12.

Expression (13) describes the distribution of the angle of the liquid crystal droplets rotation in the PDLC due to the HDS formation. This is a starting point for calculating the perturbation of the dielectric tensor $\langle \Delta {\hat{\varepsilon }}_{lc}^{pol}(r,t)\rangle$.

Solving equations (12), (13) relatively to $\varphi_{E}^{}(r,t)$ makes it possible to determine the angle of the director of molecules (droplets) rotation in a sample. Then the expression for the director itself has the following form [32]:

(15) $$C(r,t)=\left[\begin{array}{c} \cos\left[\varphi_{E}^{}(r,t)\right]\\ \sin\left[\varphi_{E}^{}(r,t)\right]\\ \sin\left[\varphi_{E}^{}(r,t)\right]\cdot \sin\left[\frac{\theta_{0}-\theta_{1}}{2}\right] \end{array}\right].$$

Thus, the perturbation of the dielectric tensor is due to the periodic change in the orientation of the molecules (droplets) of the liquid crystal and can be determined as follows:

(16) $$\mathrm{for}\;\mathrm{PSLC}\;–\;\Delta {\hat{\varepsilon }}_{lc}^{pol}(r,t)=(\varepsilon_{lc}^{o}-\varepsilon_{lc}^{e})\cdot C(r,t)C(r,t),$$

(17) $$\mathrm{for}\;\mathrm{PDLC}\;–\;\langle \Delta {\hat{\varepsilon }}_{lc}^{pol}(r,t)\rangle =(\varepsilon_{lc}^{o}-\varepsilon_{lc}^{e})\cdot \int_{0}^{\pi }\int_{0}^{\pi }C(r,t)C(r,t)p(\alpha )q(\phi )d\alpha d\phi .$$

Since the perturbations of the dielectric tensor (16, 17) are periodic, they can be represented as a Fourier series in terms of spatial harmonics:

(18) $$\mathrm{for}\;\mathrm{PSLC}\;–\;\Delta {\hat{\varepsilon }}_{lc}^{pol}(r,t)=\sum_{i=0}^{H}{\Delta {\hat{\varepsilon }}_{i}^{pol}\left(r,t\right)|}_{lc}\cdot \cos(i\cdot K\cdot r),$$

(19) $$\mathrm{for}\;\mathrm{PDLC}\;–\;\langle \Delta {\hat{\varepsilon }}_{lc}^{pol}(r,t)\rangle =\sum_{i=0}^{H}\langle {\Delta {\hat{\varepsilon }}_{i}^{pol}\left(r,t\right)|}_{lc}\rangle \cdot \cos(i\cdot K\cdot r),$$

where ${\Delta {\hat{\varepsilon }}_{i}^{pol}\left(r,t\right)|}_{lc}=\frac{1}{2\pi }\int_{-\pi }^{\pi }\Delta {\hat{\varepsilon }}_{lc}^{}\left(r,t\right)\cos(i\cdot K\cdot r)\;d(K\cdot r)$—harmonics amplitudes for PSLC and $\langle {\Delta {\hat{\varepsilon }}_{i}^{}\left(r,t\right)|}_{lc}\rangle =\frac{1}{2\pi }\int_{-\pi }^{\pi }\langle \Delta {\hat{\varepsilon }}_{lc}^{}\left(r,t\right)\rangle \cos(i\cdot K\cdot r)\;d(K\cdot r)$—harmonics amplitudes for PDLC; $K=k_{0}-k_{1}$.

### 2.2. Photopolymerization-diffusion Recording Mechanism

If in Expression (5), the vectors $E_{{}_{j}}^{m}$ are not orthogonal, then in the media a periodic interference pattern will be observed, the intensity distribution of which can be represented as [31]:

(20) $$I\left(r,t\right)=\sum_{m=o,e}I_{}^{m}\left(r,t\right)\cdot \left[1+m_{}^{m}\left(r\right)\cos\left(K_{}^{m}\cdot r\right)\right],$$

where $m_{}^{m}\left(r,t\right)=2\sqrt{I_{0}^{m}\left(r,t\right)\cdot I_{1}^{m}\left(r,t\right)}\cdot \left(e_{0}^{m}\cdot e_{1}^{m}\right)/\left(I_{0}^{m}\left(r,t\right)+I_{1}^{m}\left(r,t\right)\right)$–local contrast of interference pattern; $I_{}^{m}\left(r,t\right)=I_{0}^{m}\left(r,t\right)+I_{1}^{m}\left(r,t\right)$–total intensity; $I_{j}^{m}\left(r,t\right)={\left|E_{j}^{m}\left(r,t\right)\right|}^{2}$; as before, *m = o* corresponds to own ordinary wave in the sample, *m = e* corresponds to own extraordinary wave in the sample.

According to [31], similar to [22,28,30], the general case of the photo-polymerization-diffusion process of a HDS formation is completely described by a system of kinetic equations:

(21) $$\frac{\partial M^{m}(r,t)}{\partial t}=\mathrm{div}\left(D_{M}^{}(r,t)\cdot \mathrm{grad}M^{m}(r,t)\right)-h\cdot {\left(I^{m}(r,t)\right)}^{k}\cdot M^{m}(r,t),$$

(22) $$\frac{\partial n_{p}^{m}(r,t)}{\partial t}=\delta n_{p}\cdot h\cdot {\left(I^{m}(r,t)\right)}^{k}\cdot M^{m}(r,t),$$

(23) $$\frac{\partial n_{lc}^{m}(r,t)}{\partial t}=\delta n_{lc}\cdot \mathrm{div}\left(D_{LC}^{}(r,t)\cdot \mathrm{grad}M^{m}(r,t)\right),$$

where $M^{m}(r,t)$–monomer concentration change rate; $n_{p}^{m}(r,t)$–change in refractive index due to photo-polymerization reaction; $n_{lc}^{m}(r,t)$–change in refractive index due to mutual diffusion of components; $D_{M}^{}(r,t)$, $D_{LC}^{}(r,t)$—diffusion coefficient of monomer and liquid crystal; *k*–parameter of the photo-polymerization process nonlinearity according to the intensity of light (typical value *k* = 0,5); $\delta n_{p}$, $\delta n_{lc}$–proportionality coefficients that determine the contribution of the photo-polymerization reaction and the diffusion process to the formation of a HDS; *h*–parameter dependent on the composition of the material [22,26]:

(24) $$h=K_{g}\cdot {\left[\frac{\alpha_{0}\beta K_{d}\tau_{0}}{K_{b}^{}}\right]}^{k},$$

where $K_{g}$, $K_{b}$–polymeric chain growth and breakage rates; $\alpha_{0}$–absorption coefficient of one dye molecule; β–initiation reaction parameter; $K_{d}$–dye concentration; $\tau_{0}$–lifetime of the excited state of the dye molecule.

Changes in the refractive index due to the photo-polymerization-diffusion recording mechanism can be found as the sum of the harmonics of the refractive indices:

(25) $$\Delta n_{p}^{m}(r,t)=\sum_{i=0}^{H}{\Delta n_{i}^{m}\left(r,t\right)|}_{p}\cdot \cos(i\cdot K^{m}\cdot r),$$

(26) $$\Delta n_{lc}^{m}(r,t)=\sum_{i=0}^{H}{\Delta n_{i}^{m}\left(r,t\right)|}_{lc}\cdot \cos(i\cdot K^{m}\cdot r),$$

where ${\Delta n_{i}^{m}\left(r,t\right)|}_{p}$, ${\Delta n_{i}^{m}\left(r,t\right)|}_{lc}$–the corresponding harmonic amplitudes, which are found by solving system (21-23) similarly to [31]:

(27) $${\Delta n_{i}^{m}\left(r,\tau \right)|}_{p}=\delta n_{p}\cdot \sum_{l=0}^{H}a_{i,l}^{m}\left(r,\tau \right)\sum_{p=0}^{H}A_{i,p}^{m}\left(r,\tau \right)\frac{\exp\left(\lambda_{p}^{m}\left(r\right)\cdot \tau \right)-1}{\lambda_{p}^{m}\left(r\right)},$$

(28) $${n_{i}^{m}\left(r,\tau \right)|}_{lc}=\delta n_{lc}\cdot i^{2}\sum_{p=0}^{H}A_{i,p}^{m}\left(r,\tau \right)\frac{\exp\left(\lambda_{p}^{m}\left(r\right)\cdot \tau \right)-1}{\lambda_{p}^{m}\left(r\right)},$$

where functional dependences of $A_{i,p}^{m}\left(r,\tau \right)$ and $\lambda_{p}^{m}\left(r\right)$ are introduced in Reference [39] and the following matrix of coefficients is presented:

(29) $$a_{i,l}^{m}\left(r,\tau \right)=-\left\{\begin{array}{cccccccc} e_{1}^{m}\left(r,\tau \right) & e_{2}^{m}\left(r,\tau \right) & e_{3}^{m}\left(r,\tau \right) & 0 & 0 & . & 0 & 0\\ 2e_{2}^{m}\left(r,\tau \right) & e_{11}^{m}\left(r,\tau \right) & e_{2}^{m}\left(r,\tau \right) & e_{3}^{m}\left(r,\tau \right) & 0 & . & 0 & 0\\ 2e_{3}^{m}\left(r,\tau \right) & e_{2}^{m}\left(r,\tau \right) & e_{1}^{m}\left(r,\tau \right) & e_{2}^{m}\left(r,\tau \right) & e_{3}^{m}\left(r,\tau \right) & . & 0 & 0\\ 0 & e_{3}^{m}\left(r,\tau \right) & e_{2}^{m}\left(r,\tau \right) & e_{1}^{m}\left(r,\tau \right) & e_{2}^{m}\left(r,\tau \right) & . & 0 & 0\\ 0 & 0 & e_{3}^{m}\left(r,\tau \right) & e_{2}^{m}\left(r,\tau \right) & e_{1}^{m}\left(r,\tau \right) & . & 0 & 0\\ . & . & . & . & . & . & . & .\\ 0 & 0 & 0 & 0 & 0 & . & e_{1}^{m}\left(r,\tau \right) & e_{2}^{m}\left(r,\tau \right)\\ 0 & 0 & 0 & 0 & 0 & . & e_{2}^{m}\left(r,\tau \right) & e_{1}^{m}\left(r,\tau \right) \end{array}\right\},$$

where $e_{1}^{m}\left(r,\tau \right)=\frac{2^{k}}{b^{m}\left(r,\tau \right)}\left(1+k(k-1){\left[m_{}^{m}\left(r,\tau \right)\right]}^{2}/4\right)$, $e_{11}^{m}\left(r,\tau \right)=\frac{2^{k}}{b^{m}\left(r,\tau \right)}\left(1+3k(k-1){\left[m_{}^{m}\left(r,\tau \right)\right]}^{2}/4\right)$, $e_{2}^{m}\left(r,\tau \right)=\frac{2^{k}}{b^{m}\left(r,\tau \right)}\frac{km_{}^{m}\left(r,\tau \right)}{2}$, $e_{3}^{m}\left(r,\tau \right)=\frac{2^{k}}{b^{m}\left(r,\tau \right)}k(k-1){\left[m_{}^{m}\left(r,\tau \right)\right]}^{2}/8$, $\tau =t/T_{M}^{m}$–relative time; $T_{M}^{m}=\frac{1}{D_{Mn}^{m}\cdot {\left|K_{}^{m}\right|}^{2}}$–characteristic time of monomer’s diffusion; $D_{Mn}^{m}$–initial diffusion coefficient.

In Matrix (29), the following parameter is introduced:

(30) $$b_{}^{m}\left(r,\tau \right)=T_{P}^{m}\left(r,\tau \right)/T_{M}^{m}\left(r\right),$$

where $T_{P}^{m}\left(r,\tau \right)=\frac{1}{h\cdot {\left(I^{m}\left(r,\tau \right)\right)}^{k}}$–characteristic time of photo-polymerization process; $T_{M}^{m}\left(r\right)=\frac{1}{D_{Mn}^{m}\cdot {\left|K_{}^{m}\left(r\right)\right|}^{2}}$–characteristic time of diffusion process.

Parameter $b_{}^{m}\left(r,\tau \right)$ (30) is a key generic parameter of photo-polymerization-diffusion recording process. Due to its definition, it takes into account all of the possible non-uniformities of amplitude and phase distributions of recording field [31].

Perturbations of the dielectric tensor caused by photo-polymerization $\Delta {\hat{\varepsilon }}_{p}^{m}\left(r,\tau \right)$ and diffusion $\Delta {\hat{\varepsilon }}_{lc}^{m}\left(r,\tau \right)$ processes, can be introduced as the sum of the spatial harmonics:

(31) $$\Delta {\hat{\varepsilon }}_{p}^{m}\left(r,\tau \right)=\sum_{i=0}^{H}{\Delta {\hat{\varepsilon }}_{i}^{m}\left(r,\tau \right)|}_{p}\cdot \cos(i\cdot K_{}^{m}r),$$

(32) $$\Delta {\hat{\varepsilon }}_{lc}^{m}\left(r,\tau \right)=\sum_{i=0}^{H}{\Delta {\hat{\varepsilon }}_{i}^{m}\left(r,\tau \right)|}_{lc}\cdot \cos(i\cdot K_{}^{m}r),$$

where:

(33) $$\begin{array}{c} {\Delta {\hat{\varepsilon }}_{i}^{m}\left(r,\tau \right)|}_{p}=2n_{p}\cdot {n_{i}^{m}\left(r,\tau \right)|}_{p}\cdot \hat{I},\\ {\Delta {\hat{\varepsilon }}_{i}^{o}\left(r,\tau \right)|}_{lc}=2n_{lc}^{o}\cdot {n_{i}^{o}\left(r,\tau \right)|}_{lc}\cdot \hat{I},\\ {\Delta {\hat{\varepsilon }}_{i}^{e}\left(r,\tau \right)|}_{lc}=2\left(n_{lc}^{o}\cdot {n_{i}^{o}\left(r,\tau \right)|}_{lc}-n_{lc}^{e}\cdot {n_{i}^{e}\left(r,\tau \right)|}_{lc}\right)\cdot CC \end{array},$$

with statistical averaging of dielectric tensor for PDLC.

## 3. Numerical Simulations and Discussion

This section contains an investigation of complicated holographic diffraction structure (HDS) formation process. Simulations are made numerically and based on theoretical model developed in Section 2.

**Figure 3:**
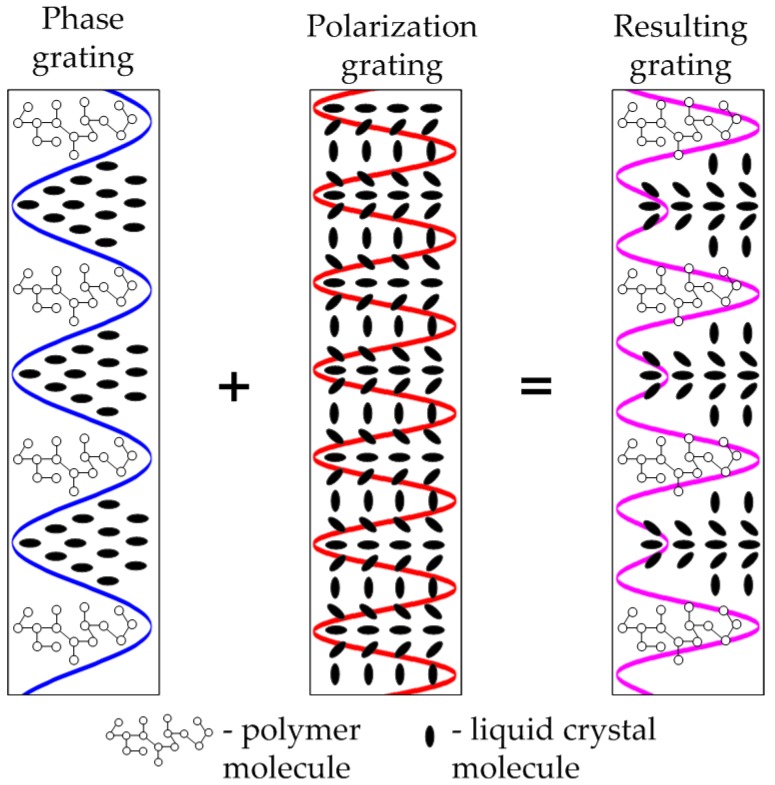
Schematic illustration of HDS being formed by arbitrary polarized waves in PDLC (PSLC).

As it was noted earlier, when polarization states of formation waves are arbitrary, diffraction structure represents the superposition of phase (formed due to diffusional separation of components) and polarization (formed due to orientational effects) diffraction gratings (Figure 3).

To compare contributions of photopolymerization-diffusion and polarization processes to final perturbation of dielectric tensor the analysis of harmonics amplitudes by solving Equations (18), (19), and (33) should be made.

The model of photo-polymerization-diffusion formation process for phase gratings formation (Figure 3) given in Section 2.2 is experimentally proved (in less general form) in [22,27,40]. Additionally, in Reference [22], the good compliance with non-local polymerization driven diffusion model (NPDD) [23,24] is shown. It have been noted that introduced parameter $b_{}^{m}\left(r,\tau \right)$ (analogue to R in NPDD model) is the key generic parameter that defines the harmonics amplitudes (27)–(30).

Generic parameter $b_{}^{m}\left(r,\tau \right)$ includes all of the physical characteristics of material and recording geometry [31]. These characteristics are (30), (24): Spatial frequency, formation field intensity, diffusion coefficients, photo-polymerization reactions rates, etc.

Analysis of Expressions (20) and (5), describing the spatial distribution of the field forming the HDS, shows that when recording a structure by arbitrarily polarized waves, the intensity of the total field differs from the maximum possible. That fact, in accordance with Reference [31], leads to a change in the parameter $b_{}^{m}\left(r,\tau \right)$. Thus, to estimate the contribution of the photo-polymerization-diffusion mechanism to the formation of a non-uniform HDS by arbitrarily polarized waves, it is sufficient to calculate the amplitudes of the harmonics with varying $b_{}^{m}\left(r,\tau \right)$.

Hereinafter, for definiteness and simplicity, the following conditions of numerical simulation are taken:

- spatial harmonics’ amplitudes are calculated for one dielectric tensor element $\Delta {\hat{\varepsilon }}_{i}^{m}{}_{}^{\left[1,1\right]}={\Delta {\hat{\varepsilon }}_{i}^{m}|}_{p}^{\left[1,1\right]}+{\Delta {\hat{\varepsilon }}_{i}^{m}|}_{lc}^{\left[1,1\right]}$ for phase and ${\Delta {\hat{\varepsilon }}_{i}^{pol}|}_{lc}^{\left[1,1\right]}$ for polarization gratings;
- calculations are made at the end of HDS formation (all of the photo-polymerization processes are finished);
- light-induced absorption coefficient change is negligible;
- intensities of formation waves are equal;
  formation light waves are flat $A_{j}^{}\left(r\right)=1$, $\varphi_{j}\left(r\right)=0$, coherent, and monochromatic.
  Taken conditions make it possible to simplify parameter $b_{}^{m}\left(r,\tau \right)=b_{}^{m}$.

Thus, $b_{}^{m}$ still define the harmonics’ amplitudes, but doesn’t depend on time and spatial coordinates.

**Figure 4:**
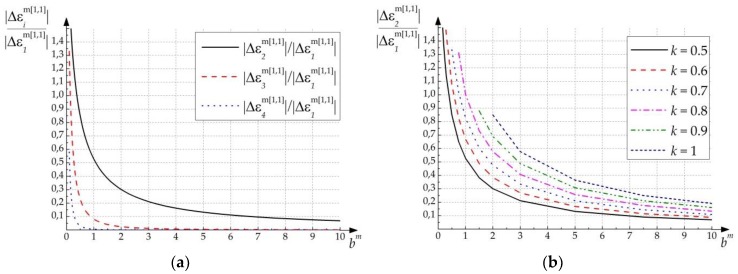
Calculated ratios: (**a**) of the first and higher harmonics depending on the parameter $b_{}^{m}$ (for *k* = 0,5); (**b**) of the first and second harmonics for different *k*.

Figure 4a contains calculated ratios of the first and higher harmonics depending on the parameter $b_{}^{m}$ (for *k* = 0,5). Figure 4b contains a family of curves describing the ratio of the first and second harmonics for different *k*.

As can be seen from Figure 4, in the case of $b_{}^{m}>1$ (k≤0.5) formation process is characterized as almost linear (it means that perturbation of dielectric permittivity is quasi-sinusoidal) in comparison with case when $b_{}^{m}<1$. HDS formed with $b_{}^{m}<1$ has a pronounced character of higher harmonics (especially the second one).

According to Expression (30), parameter $b_{}^{m}$ decreases due to following reasons:

- changing material’s parameters;
- decreasing spatial frequency of HDS (in this case value ${\left|K_{}^{m}\right|}^{2}$ is reduced and characteristic time $T_{M}^{m}$ increases);
- increasing formation field’s intensity (in this case characteristic time $T_{P}^{m}$ is reduced).

Two of three given reasons are defined by recording conditions. Thus, when recording process occurs with high light field’s intensities and with big spatial period of interference pattern (for example, angles of incidence for transmission grating are small), second spatial harmonic will be rather high.

Experimental works describing polarization gratings in PDLC (PSLC), such as Reference [36], note that recording should be held at small incident angles. Besides, intensity of formation waves should be high to cross the threshold of Freedericksz transition [35,38]. Thus, described conditions of polarization gratings’ formation correspond to conditions of non-linear phase gratings formation.

To study more general cases of the formation of a polarization grating, it is advisable to calculate the amplitudes of the harmonics with varying parameters of the state of polarization of the forming field. Before doing it, some physical parameters should be defined for calculations: $K_{33}=7.45\cdot 10^{-6}$ din; R=0.1 um; $W_{a}=10^{-5}$; δ=0.1; $\left|E(r,t)\right|=1.5\cdot E_{c}$; $E_{c}$–critical electric field strength.

**Figure 5:**
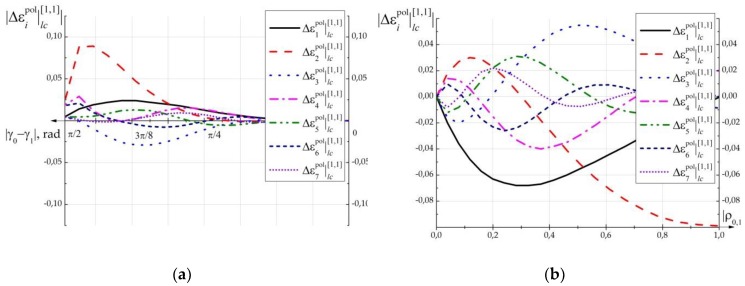
Amplitudes of harmonics of dielectric permittivity perturbation for polarization grating formed in PSLC: (**a**) As a function of an angle between polarization vectors of two orthogonally linearly polarized formation beams; (**b**) as a function of the ellipticity of the two formation beams with orthogonal elliptical polarization $\rho_{0}=-\rho_{1}$.

Figure 5 contains results of calculation of first seven harmonics ${\Delta {\hat{\varepsilon }}_{i}^{}|}_{lc}^{\left[1,1\right]}$ for polarization grating formed in PSLC as a function of an angle between polarization vectors of two orthogonally linearly polarized formation beams $\left|\gamma_{0}-\gamma_{1}\right|$. Figure 5b shows the dependence of the amplitudes of the polarization grating (formed in PSLC) harmonics on the ellipticity of the two formation beams with orthogonal elliptical polarization.

From Figure 5a it can be seen that the maximum change in the dielectric constant of the PSLC (PDLC), caused by the recording of HDS, is observed at polarization states of linearly polarized waves close to orthogonal. In the case when $\left|\gamma_{0}-\gamma_{1}\right|=0$ the HDS recording does not occur.

From Figure 5b it can be seen that the different degree of ellipticity of polarized formation beams causes the formation of complex inhomogeneous HDS. In the case of $\rho_{0}=1$, $\rho_{1}=-1$, the structure has the most pronounced harmonic character with the predominance of the second and fourth harmonics.

Analysis of Figure 5 shows that formed polarization gratings have a non-harmonic character with a pronounced predominance of even harmonics. The predominance of even harmonics (in particular the second one) in the structure of polarization gratings can be mathematically explained by the presence of the product C(r,t)C(r,t) in Expressions (16, 17), which causes the influence of squares $\cos^{2}\left[\varphi_{E}^{}(r,t)\right]$ and $\sin^{2}\left[\varphi_{E}^{}(r,t)\right]$ on tensor perturbation. Physically, this effect can be explained by the symmetry of the LC molecules with respect to the axis of their rotation. Therefore, the change in dielectric constant when the molecule is rotated by a positive angle will be similar to the change when it is rotated to a negative angle, and thus, the period of $\Delta {\hat{\varepsilon }}_{lc}(r,t)$ change doubles. Additionally, doubled period of polarization gratings in PDLC is shown experimentally in [36] and another experimental works.

Additionally, it can be seen that recording by two waves with orthogonal circular polarization leads to the formation of polarization grating with a more pronounced harmonic character compared to the structure recorded with linear orthogonal polarized waves, since in this case there is no modulation of ellipticity. However, in this case, even harmonics also make the main contribution.

Analysis of formation of polarization gratings in PDLC shows effects similar to formation in PSLC with smoothly decreasing character of amplitudes of even harmonics’ behaviour.

Figure 4 and Figure 5 show that when intensity of the recording field exceeding the critical value ($\left|E(r,t)\right|>E_{c}$), the perturbation of the dielectric tensor caused by the phase grating formation can be commensurate with the perturbation caused by the polarization grating formation in the same sample. This effect will be especially vivid in the case of polarization states of recording beams that are close to orthogonal.

To confirm this conclusion, a quantitative estimate of the second harmonic’s amplitude was made by expression (33), based on the following conditions: Average refractive index of the composition n=1.54; $\delta n_{p}\approx 0.004$; $\delta n_{lc}=0.04$ (obtained experimentally in References [40,41]) amplitude of the second harmonic of the refractive index $n_{2}^{e}=0.35$. Then, $\Delta {\hat{\varepsilon }}_{2}^{m}\approx 2\cdot 1.54\cdot 0.04\cdot 0.35=0.043$. In this case, the amplitude of the second harmonic of the polarization grating (see Figure 5) is within $\Delta {\hat{\varepsilon }}_{2}^{pol}=0.005÷0.1$. The evaluation shows the validity of the conclusion about the proportionality of the contributions of the two mechanisms in the formation of diffraction structures.

## 4. Conclusions

In this paper we developed an analytical model for the HDS formation in PSLC and PDLC, which takes into account an arbitrary polarization state of recording beams. The forms of spatial profiles of perturbations of the dielectric tensor are numerically investigated. It is shown that the main contribution to the perturbation of the dielectric tensor of a material when only the polarization mechanism is taken into account is made by even spatial harmonics of changes in the dielectric permittivity (in particular, the second one).

It is shown that the perturbation of the dielectric permittivity in the process of recording of polarization gratings is comparable in magnitude with the perturbation caused by recording phase gratings in this material. It suggests the need to take into account the polarization mechanism during holographic recording by arbitrarily polarized beams.
